# In the thick of it: radish thermotolerance and root development under heat shock

**DOI:** 10.1093/plphys/kiag060

**Published:** 2026-02-06

**Authors:** Héctor H Torres-Martínez

**Affiliations:** Assistant Features Editor, Plant Physiology, American Society of Plant Biologists, Rockville, United States; Howard Hughes Medical Institute, Stanford University, Stanford, CA 94305, United States

One of the most evident consequences of contemporary climate change is the rise in global mean temperature, a phenomenon that negatively affects crop yields at both global and local scales (Zhao et al. 2017). Among abiotic stresses, heat stress is particularly detrimental to plant growth, impairing cell division and expansion through both passive and active mechanisms. Passive inhibition reflects the general slowing of cellular processes under suboptimal conditions, whereas active inhibition involves stress-triggered signaling pathways that promote adaptive responses to unfavorable environments (Zhang et al. 2020).

Heat stress induces extensive reprogramming of root cells through mechanisms operating at multiple regulatory levels, including complex hormone crosstalk involving auxin, cytokinin, and abscisic acid (ABA), ultimately leading to root developmental adaptations (Tiwari et al. 2022). Early signaling events such as calcium (Ca²⁺) influx play a central role in the heat shock response by activating downstream HEAT SHOCK PROTEINS (HSPs) and HEAT SHOCK FACTORS (HSFs). In parallel, ABA signaling promotes the accumulation of hydrogen peroxide (H₂O₂), accompanied by the activation of reactive oxygen species (ROS)–scavenging enzymes, thereby enhancing thermotolerance. Similar to Ca²⁺ signaling, ABA has also been shown to regulate HSP expression during heat stress (Tiwari et al. 2022).

In root crops, storage root yield largely depends on vascular cambium development, known as secondary growth, which occurs primarily in the hypocotyl and the upper portion of the taproot (Hoang, Park, et al. 2020). High temperatures have been reported to impair storage root initiation in sweet potato (Gajanayake et al. 2014). In radish (*Raphanus sativus*), numerous differentially expressed genes, including transcription factors from diverse families, respond to heat stress (Wang et al. 2018). Initial efforts to link heat stress-responsive genes with root developmental regulation in root crops were carried out using tissue-specific transcriptomics in radish (Hoang et al. 2020). However, the complexity of the regulatory networks integrating secondary growth and heat stress responses has remained largely unresolved.

In this issue of *Plant Physiology*, Li et al. (2026) identified a genetic module that integrates heat stress signaling with taproot development in radish. The authors demonstrated that the transcription factors RsWRKY18 and RsHSFA2 act cooperatively to enhance the expression of the heat stress-responsive gene *RsHSP22*, as well as several genes associated with taproot thickening, including *RsXTH32*, *RsEXPA9*, *RsWOX14*, and *RsKNAT1* (Fig. 1).

**Figure 1:**
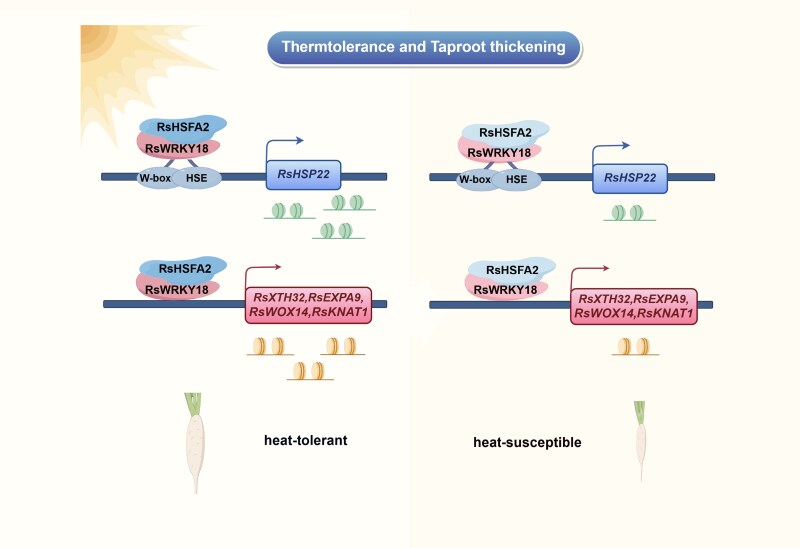
**A proposed working model of the RsWRKY18 interacting with RsHSFA2 activates RsHSP22 and taproot thickening-related genes in response to high temperature in radish.** When radish suffers from high temperature, RsWRKY18 and RsHSFA2 are rapidly induced, and binds to the RsHSP22 promoter to activate its expression. RsWRKY18 could bind to the promoters of taproot thickening-related genes including RsXTH32, RsEXPA9, RsKNAT1 and RsWOX14. RsHSFA2 interacts with RsWRKY18 coordinately to enhance the transcription activity of RsHSP22 and RsWRKY18-target genes, especially under HS conditions, thereby activating radish thermotolerance and taproot thickening. The difference of transcription level of RsHSFA2 and RsWRKY18 between two radish genotypes under high temperature differentiates the heat tolerances. This diagram was drawn by Figdraw. The green circles represent the transcript of RsHSP22 gene, and the brown circles indicate the transcript of taproot thickening-related genes. Figure and caption from highlighted article (Li et al., 2026).

Using 2D gel electrophoresis coupled with mass spectrometry, the authors identified a protein specifically induced in radish roots at 12 and 24 h after heat shock treatment (40 °C), which was identified as HEAT SHOCK PROTEIN 22 (RsHSP22). Consistently, *RsHSP22* transcript levels increased following heat stress and were significantly higher in a heat-tolerant genotype than in a heat-sensitive one. Constitutive expression of a chimeric *RsHSP22-GFP* construct in *Arabidopsis thaliana* resulted in enhanced survival under heat shock compared to wild-type plants, suggesting that RsHSP22 functions as a positive regulator of thermotolerance. Moreover, the mitochondrial localization of RsHSP22-GFP supports its functional role in heat stress responses.

Analysis of the *RsHSP22* promoter revealed multiple W-box elements, which are binding sites for WRKY transcription factors, as well as a heat shock element (HSE) recognized by HSFs. This promoter architecture prompted the researchers to explore *RsHSP22* transcriptional regulation. Transient expression assays, using either luciferase or ß-glucuronidase as reporters, in tobacco leaves showed that truncated versions of the *RsHSP22* promoter containing only a single W-box and a mutated HSE led to a marked reduction in reporter activity. In contrast, the full-length promoter resulted in strong reporter expression under heat stress conditions.

To identify transcription factors binding the *RsHSP22* promoter, Li et al. examined radish transcriptomic datasets and found that *RsWRKY18* is expressed in root tissues, while *RsHSFA2* is strongly induced by heat stress. Yeast 1-hybrid assays confirmed that RsWRKY18 and RsHSFA2 bind directly to the *RsHSP22* promoter, targeting W-boxes (W1 and W3) and the HSE, respectively. Dual-luciferase assays demonstrated that either transcription factor alone enhanced *RsHSP22* promoter activity, as long as their respective *cis*-elements were intact.

RT-qPCR analyses further showed that both *RsWRKY18* and *RsHSFA2* transcripts accumulated in radish roots under heat stress, with higher expression levels in the heat-tolerant genotype. Consistently, promoter-reporter assays confirmed that the activities of both promoters increased in response to heat shock. Overexpression of *RsWRKY18* or *RsHSFA2* in *Arabidopsis* resulted in improved survival under heat stress, while radish plants overexpressing *RsWRKY18* exhibited reduced wilting and lower ROS accumulation, supporting a conserved role for these factors in thermotolerance.

Given their similar expression patterns and shared regulation of *RsHSP22*, the authors tested whether RsWRKY18 and RsHSFA2 physically interact and act synergistically. Yeast 2-hybrid and pull-down assays confirmed direct interaction between the 2 proteins. Transient co-expression of *35S-RsWRKY18* and *35S-RsHSFA2* in radish cotyledons led to reduced ROS accumulation under heat stress compared to individual expression. Electrophoretic mobility shift assays showed that RsWRKY18 enhances RsHSFA2 binding to the *RsHSP22* promoter, and co-expression of both factors strongly increased *RsHSP22* promoter activity in dual-luciferase assays, particularly under heat shock. Together, these results demonstrated that RsWRKY18 and RsHSFA2 form a cooperative transcriptional module regulating *RsHSP22* expression.

Virus-induced gene silencing of *RsWRKY18* in radish resulted in more severe wilting after heat stress, accompanied by reduced root weight, thinner taproots, increased ROS accumulation, and decreased *RsHSP22* expression. In *Arabidopsis*, *RsWRKY18* overexpression promoted primary root elongation and increased root apical meristem activity, indicating a role in root development. Heat stress reduced vascular cambium cell layers and root weight in both heat-tolerant and sensitive radish genotypes, effects that were exacerbated by *RsWRKY18* silencing.

Radish transcriptomic analyses revealed that root thickening-related genes such as *XTH32*, *EXPA9*, *WOX14*, and *KNAT1* are highly expressed in roots and contain W-box elements in their promoters. Yeast one-hybrid and dual-luciferase assays supported direct regulation of these genes by RsWRKY18. Furthermore, heat stress and *RsWRKY18* overexpression induced root thickening-related genes expression, whereas *RsWRKY18* silencing led to their downregulation. Finally, co-expression of *RsWRKY18* and *RsHSFA2* further enhanced the activation of these developmental genes, particularly under heat stress.

Collectively, Li et al. (2026) uncover a transcriptional module that links heat stress responses with taproot thickening in radish (Fig. 1). This work significantly advances our understanding of storage root development under adverse temperatures and provides a promising framework for improving thermotolerance and yield in root crops through targeted genetic strategies.

## Data Availability

No new data were generated or analysed in support of this article.
